# Commentary: A Humanized Clinically Calibrated Quantitative Systems Pharmacology Model for Hypokinetic Motor Symptoms in Parkinson's Disease

**DOI:** 10.3389/fphar.2016.00179

**Published:** 2016-06-20

**Authors:** Patricia Muñoz, Juan Segura-Aguilar

**Affiliations:** Molecular and Clinical Pharmacology, Faculty of Medicine, University of ChileSantiago, Chile

**Keywords:** preclinical models, neuromelanin, dopamine oxidation, aminocromo, neuronal dysfunction, mitochondrial dysfunction, protein degradation dysfunction, alpha-synuclein oligomers

This publication proposes a computer-based platform to explore novel targets for symptomatic treatment, based on the known neuroanatomy and neurophysiology of the basal ganglia (Roberts et al., 2016). However, this theoretical proposal should also take into consideration other aspects, such as the possible molecule(s) involved in the loss of dopaminergic neurons containing neuromelanin, because there is a long list of molecules that failed to translate successful preclinical to clinical studies and new therapies.

The discovery that PD motor symptoms were linked to the loss of dopaminergic neurons containing neuromelanin was a very important input in the search for new pharmacological treatments and understanding the mechanism underlying the degeneration of the nigrostriatal system (Fahn, 2015). This discovery also had a great influence on preclinical models that were developed by using exogenous neurotoxins that induce the degeneration of nigrostriatal neurons. The first exogenous neurotoxins used as a preclinical model of PD were 6-hydroxydopamine and later1-methyl-4-phenyl-1,2,3,6-tetrahydropyridine (MPTP) and rotenone. For decades, these preclinical models have been used both to study the molecular mechanisms of the disease and to test new possible drugs or treatments. A long list of drugs and even gene therapy has been tested with these preclinical models, showing successful results, but these failed in clinical studies (Athauda and Foltynie, 2015; Lindholm et al., 2016; Olanow et al., 2015; Park and Stacy, 2015).

In our opinion, the failure to translate the successful results obtained in preclinical studies to clinical studies is not a problem of incorrect targets but depends on (i) the identity of the neurotoxin causing the loss of dopaminergic neurons, containing neuromelanin in the substantia nigra, which remains unknown. The discovery of genes associated with a familial form of PD provided an enormous input to the basic research into understanding the mechanism involved in the degeneration of the nigrostriatal system. However, it is unclear what induces the dysfunction of these genes in the sporadic form of disease. There is a general agreement in the scientific community that the degeneration of the nigrostriatal neurons in PD involves mitochondrial dysfunction, aggregation of alpha-synuclein to neurotoxic oligomers, dysfunction of protein degradation, oxidative stress, neuroinflammation and endoplasmic reticulum stress (Segura-Aguilar et al., 2014, 2016a) and (ii) preclinical models that do not reflect what is happening in the disease since these models are based on exogenous neurotoxins that do not exist in dopaminergic neurons. These models have been used both to study the mechanisms of neurodegeneration and to test new drugs. The degenerative process of the nigrostriatal system in Parkinson's disease is extremely slow because it takes years for motor symptoms to become evident. This contrasts with the extremely rapid and extensive degenerative process of the nigrostriatal system induced by exogenous neurotoxins such as 6-hydroxydopamine, MPTP or rotenone. The best example is the MPTP that induces severe Parkinsonism in humans just 3 days after the consumption of drugs contaminated with this neurotoxin (Segura-Aguilar et al., 2014, 2016b). The exogenous neurotoxin models have been very useful as models for studies of mechanisms for neurodegeneration (Segura-Aguilar and Kostrzewa, 2015) but they have been completely worthless as preclinical models for Parkinson's disease (Athauda and Foltynie, 2015; Lindholm et al., 2016; Olanow et al., 2015; Park and Stacy, 2015; Segura-Aguilar et al., 2016a,b).

It has been proposed that aminochrome, a metabolite of dopamine oxidation to neuromelanin, can be both used in a preclinical model for Parkinson's disease and also represents an endogenous neurotoxin that triggers the loss of dopaminergic neurons containing neuromelanin in the substantia nigra (Segura-Aguilar et al., 2014, 2016a; Herrera et al., 2016). Recently, it has been reported that the unilateral injection of aminochrome into the striatum induced a dysfunction of dopaminergic neurons characterized by (i) an imbalance between the level of dopamine and GABA as a consequence of lower release of dopamine; (ii) induction of a progressive contralateral behavior without significant loss of the nigrostriatal system; (iii) induction of mitochondrial dysfunction resulting in lower levels of ATP required for both axonal transport of synaptic vesicles and dopamine release; (iv) a significant decrease in the number of synaptic vesicles in the terminals; and (v) the induction of morphological changes in dopaminergic neurons (cell shrinkage) (Herrera et al., 2016).

The difference between aminochrome and exogenous neurotoxins used in the preclinical model of Parkinson's disease is that aminochrome is produced within dopaminergic neurons lost during Parkinson's disease and does not induce a rapid and massive loss of the nigrostriatal system, but induces a progressive dysfunction of dopaminergic neurons based on an imbalance between neurotransmitters, as in Parkinson's disease. Rats fed β-sitosterol β-d-glucoside have been proposed as a new preclinical model of Parkinson's disease and the question is whether this compound is generated inside dopaminergic neurons (Van Kampen et al., 2015). Aminochrome has been proposed as the endogenous neurotoxin that triggers the loss of dopaminergic neurons containing neuromelanin, since aminochrome induces mitochondrial dysfunction (Arriagada et al., 2004; Paris et al., 2011; Aguirre et al., 2012; Muñoz et al., 2012), protein degradation dysfunction (Zafar et al., 2006; Huenchuguala et al., 2014), endoplasmic reticulum stress (Xiong et al., 2014), oxidative stress (Arriagada et al., 2004) and the formation of neurotoxic oligomers of alpha-synuclein (Muñoz et al., 2015).

Dopamine oxidation to neuromelanin is a normal pathway since healthy individuals have intact dopaminergic neurons containing neuromelanin in the substantia nigra (Segura-Aguilar et al., 2014). The reason why aminochrome is not neurotoxic in healthy individuals is because two enzymes [DT-diaphorase and glutathione transferase M2-2 (GSTM2)] prevent aminochrome-induced neurotoxicity (Segura-Aguilar et al., 2014, 2016a). DT-diaphorase is expressed in dopaminergic neurons and astrocytes. GSTM2 is only expressed in astrocytes, but astrocytes secrete GSTM2 into the conditioned medium to protect dopaminergic neurons against aminochrome-induced neurotoxicity as these neurons are able to internalize GSTM2 (Cuevas et al., 2015; Segura-Aguilar, 2015; Segura-Aguilar et al., 2016a).

In conclusion, a new computer platform to explore new targets for new drugs is very important, but we also need to understand why we cannot translate successful results from preclinical to clinical studies to develop new pharmacological therapies.

## Author contributions

All authors listed, have made substantial, direct and intellectual contribution to the work, and approved it for publication.

## Funding

Supported by ENLACE, University of Chile.

### Conflict of interest statement

The authors declare that the research was conducted in the absence of any commercial or financial relationships that could be construed as a potential conflict of interest. The reviewer FM and handling Editor declared their shared affiliation, and the handling Editor states that the process nevertheless met the standards of a fair and objective review.
